# A New Gain-of-Function NLRP1 Variant Is Associated with Inflammasome Activation and Disease Severity in SLC25A38 Congenital Sideroblastic Anemia Case Report

**DOI:** 10.3390/jpm16090471

**Published:** 2026-09-15

**Authors:** María Sánchez-Villalobos, Eulalia Campos Baños, Elena Martínez-Balsalobre, Miriam Pinilla-Marquínez, Verónica Navarro-Ramírez, Thomas Macartney, Laura Hurtado-Navarro, David Hernández-García, Miguel Blanquer, Pablo Pelegrín, Eduardo Salido-Fierrez, Ana Belén Pérez-Oliva

**Affiliations:** 1 Biomedical Research Institute of Murcia (IMIB-Pascual Parrilla)-FFIS, 30120 Murcia, Spain; maria.sanchez74@carm.es (M.S.-V.); eulalia.camposb@um.es (E.C.B.); elena.martinez20@um.es (E.M.-B.); miriam.pinilla@imib.es (M.P.-M.); veronica.navarro@imib.es (V.N.-R.); laura.hurtado1@um.es (L.H.-N.); d.hernandezgarcia@um.es (D.H.-G.); miguel.blanquer@carm.es (M.B.); pablopel@um.es (P.P.); eduardoj.salido@carm.es (E.S.-F.); 2 MRC Protein Phosphorylation and Ubiquitylation Unit, College of Life Sciences, University of Dundee, Dundee DD1 4HN, UK; t.j.macartney@dundee.ac.uk; 3 Hematology Service, Virgen de la Arrixaca University Hospital, 30120 Murcia, Spain; 4 Department of Biochemistry and Molecular Biology B and Immunology, Faculty of Medicine, University of Murcia, 30120 Murcia, Spain

**Keywords:** case report, sideroblastic congenital anemia, NLRP1 inflammasome, erythropoiesis

## Abstract

Inflammasomes play a pivotal role in inflammation and infection, but the clinical relevance of inflammasomes goes beyond infectious disease. Here, we report a novel variant in the NLRP1 gene (p.V939M) identified in a patient with congenital sideroblastic anemia due to a homozygous SLC25A38 mutation. This patient exhibits severe anemia, iron overload, and dependence on frequent transfusions. Functional characterization of peripheral blood mononuclear cells from the patient revealed constitutive activation of the NLRP1 inflammasome in primary cells, evidenced by increased processing of NLRP1, cleavage of CASP1, and ASC oligomerization. Moreover, in a speck in vitro assay, the newly identified genetic variant increases ASC oligomerization, and using ex vivo erythroid differentiation assays with patient-derived cells, we demonstrated that pharmacological inhibition of CASP1 enhances erythrocyte formation. This patient-derived NLRP1 variant was evaluated in a newly generated human NLRP1 knockdown system using CRISPR-Cas9 in K562 cells, in combination with ferroptotic/oxidative stress induction to mimic patient clinical features, and our results indicate that the presence of NLRP1 p.V939M, together with enhanced ferroptosis, may contribute to the patient’s phenotype. Furthermore, both CASP1 inhibition and ferroptosis inhibition further enhance the restoration of erythroid colony formation from primary progenitor cells derived from the patient. Therefore, our study identifies a novel NLRP1 inflammasome variant involved in human erythropoiesis and highlights its possible contribution to the pathogenesis of congenital sideroblastic anemia, providing a basis for the development of new potential therapeutic strategies.

## 1. Introduction

Congenital sideroblastic anemias (CSAs) are a heterogeneous group of inherited disorders characterized by ineffective erythropoiesis and the presence of ring sideroblasts in the bone marrow, defined as erythroid precursors with pathological mitochondrial iron accumulation surrounding the nucleus [1]. CSAs are caused by mutations in genes involved in heme biosynthesis, iron–sulfur cluster biogenesis, mitochondrial protein synthesis, or oxidative phosphorylation pathways [2]. These defects impair erythroid maturation and hemoglobin production, leading to varying degrees of anemia and systemic iron overload. Clinically, patients commonly present with hypochromic microcytic anemia, fatigue, pallor, hepatosplenomegaly, and progressive iron accumulation secondary to ineffective erythropoiesis and chronic transfusion requirements [3].

The most common forms of CSA are caused by mutations in *ALAS2* and *SLC25A38*, both involved in heme biosynthesis within erythroid mitochondria. While *ALAS2*-associated disease is often responsive to pyridoxine supplementation and usually presents with milder phenotypes, *SLC25A38* deficiency represents one of the most frequent and severe forms of CSA [3]. *SLC25A38* encodes a mitochondrial glycine transporter required for δ-aminolevulinic acid (ALA) synthesis, an essential step in heme production. Patients carrying recessive loss-of-function variants in *SLC25A38* typically develop severe transfusion-dependent microcytic anemia from early infancy, profound systemic iron overload, and poor response to conventional therapies, making hematopoietic stem cell transplantation the only curative option currently available [4]. In addition to *ALAS2* and *SLC25A38*, mutations in several other genes, including *SLC19A2*, *GLRX5*, and *ABCB7*, have also been associated with CSA pathogenesis [5].

Mitochondrial iron overload and defective heme synthesis generate a cellular environment characterized by oxidative stress and reactive oxygen species (ROS) accumulation. These alterations have recently been linked to ferroptosis, an iron-dependent form of regulated cell death driven by lipid peroxidation [6]. Unlike apoptosis or necrosis, ferroptosis is tightly associated with intracellular iron metabolism and mitochondrial dysfunction, both hallmark features of sideroblastic anemia. Indeed, ferroptosis has been implicated in erythroid failure in CSA models, particularly in *ALAS2*-associated disease [7]. However, the contribution of inflammatory cell death pathways to the pathophysiology of CSA remains poorly understood.

Increasing evidence suggests that oxidative stress and iron dysregulation can promote activation of inflammasomes, cytosolic multiprotein complexes involved in innate immune sensing and inflammatory signaling. Inflammasome activation results in the cleavage of pro-Caspase-1 (pro-CASP1) into active CASP1, leading to the maturation of the pro-inflammatory cytokines IL-1β and IL-18, as well as the induction of pyroptosis, a highly inflammatory form of programmed cell death [8,9]. ROS accumulation has been shown to activate inflammasome pathways, including NLRP3 signaling, and to modulate Gasdermin D activation [10,11], suggesting a potential mechanistic link between ferroptosis-associated oxidative stress and inflammasome-mediated inflammation.

Inflammasomes are multiprotein complexes composed of sensor proteins such as NLRP1, NLRP3, NLRC4, and AIM2, together with the adaptor protein ASC and the effector protease CASP1 [12]. Although inflammasomes are essential for antimicrobial defense, their dysregulation has been implicated in autoinflammatory, autoimmune, metabolic, neurodegenerative, and hematological disorders [13,14,15,16].

Among the inflammasome sensors, NLRP1 remains comparatively understudied due to major structural and functional differences between human and murine orthologs [17]. Human NLRP1 contains a pyrin domain (PYD), which is absent in the murine ortholog, a NACHT domain, a leucine-rich repeat (LRR) region, a FIIND domain, and a C-terminal CARD domain. Activation critically depends on autoproteolytic cleavage within the FIIND domain, enabling release of the active C-terminal fragment and downstream inflammasome assembly [18,19]. Interestingly, independent studies in zebrafish and murine systems have suggested a role for NLRP1 signaling in hematopoiesis and erythroid regulation [20,21].

Here, we describe for the first time the involvement of inflammasome dysregulation in SLC25A38-associated congenital sideroblastic anemia through the identification of a novel NLRP1 gain-of-function variant, p.V939M, in a patient with severe disease. We demonstrate that this variant enhances inflammasome activation and could contribute to disease severity. Functional studies in NLRP1 knockout cellular systems re-expressing p.V939M NLRP1 under ferroptotic stress conditions revealed increased inflammasome activity associated with the novel variant. Furthermore, primary patient-derived cells showed that combined inhibition of ferroptosis and CASP1 activity improved erythropoiesis, supporting a pathogenic interplay between ferroptosis and inflammasome activation in this CSA patient.

## 2. Materials and Methods

### 2.1. Cell Culture

K562 cells (CRL-3343; American Type Culture Collection) were maintained in RPMI culture medium supplemented with 10% fetal calf serum (FCS), 2 mM glutamine, and 1% penicillin–streptomycin (Life Technologies, Carlsbad, CA, USA). They were tested for mycoplasma contamination and authenticated by STR profiling. Cells were maintained and subcultured before confluence every 72 h. For differentiation, cells were treated with 50 μM hemin (#16009-13-5, Sigma-Aldrich, St. Louis, MO, USA), prepared as previously described [22], in the presence of 0.1% DMSO alone or with the CASP1 inhibitor VX-765 (100 μM) (MedChemExpress, Monmouth Junction, NJ, USA). Cells were collected at different time points (0, 24, 48, 72, 96 h post-hemin addition), centrifuged, washed twice with PBS, and stored at −80 °C for further analysis.

HEK293 cells that stably express ASC GFP (donated by Dr. E. Meunier) were maintained in DMEM medium supplemented and cultivated under the same conditions as the K562 cells.

### 2.2. Primary Cell Collection and Erythroid Differentiation

Blood samples were obtained from several healthy donors or a CSA patient provided by the Hematology Service at Clinic University Hospital Virgen de la Arrixaca (HCUVA, Murcia, Spain) with ethics approval number (2021-11-9-HCUVA; 2023-3-5-HCUVA). Also consent for publication was signed and is managed by physicians. PBMCs were purified using standard Ficoll-Paque (Cytiva, Marlborough, MA, USA) gradient centrifugation. Cells were plated in duplicate at a density of 1 million/well in 6-well plates and incubated and cultured at 37 °C in a humidified atmosphere with 5% CO_2_ from day 0 to day 15. The medium used to grow the cells is Iscove modified Dulbecco medium (Gibco, Grand Island, NY, USA, 12440-053) containing 3% human AB serum (Atlanta Biologicals, Flowery Branch, GA, USA, S40110), 2% human AB plasma (SeraCare, Milford, MA, USA, 501973), 100 U/mL penicillin, 100 mg/mL streptomycin (Gibco), 200 µg/mL human holotransferrin (Sigma-Aldrich, T0666-1G), 10 µg/mL recombinant human insulin (Sigma-Aldrich, I9278), 3 IU/mL heparin (ReigJofre, Barcelona, Spain, 608737.4), 10 ng/mL human stem cell factor (PeproTech, Cranbury, NJ, USA, 300-07), 1 ng/mL interleukin 3 (IL-3) (PeproTech 200-03), and 3 IU/mL erythropoietin (Gibco PHC2054). Cells were collected from day 0 to day 12, rinsed once with PBS 1×, and the pellet was frozen at −80 °C before protein quantification and immunoblotting.

### 2.3. CRISPR-Cas

To perform the NLRP1 knockout in K562 cells, the NEPA21 electroporator was used. Cells were washed with Optimem to remove excess serum and traces of antibiotics before electroporation, and no antibiotics were used during the electroporation process or immediately following electroporation. Electroporation was established at 200 V, 3.0 ms, yielding 80% positive cells. For K562 cells, 1 × 10^6^ was used in 100 µL of Opti-MEM. Cas9 D10A nickase and guides were from IDT.

Guides—IDT 2nM alt-R—were resuspended in 20 µL duplex buffer from a 100 µM stock. Two independent pairs targeting Exon 2 of NLRP1 were used (Left A ccaccgcagtgctaatgccc and Right A accccaggtggggttcactt/Left B ctggatccatgaattgccgg and Right B attagcactgcggtggaggt). After electroporation, Western blots were performed, and the optimal pool (2.2) was sorted in 96-well plates, and clones were selected by puromycin. After clones’ growth, another Western blot analysis was performed to select the positive clones.

### 2.4. Perls Staining

Cells were fixed with methanol for 10–15 min and left to air-dry overnight. Then, samples were incubated with a potassium ferrocyanide and hydrochloric acid solution (1:1) for 2 h at 37 °C. Samples were washed with water and left to air-dry for 30 min, incubated in Harris Safranin for 20 min, and washed with water.

### 2.5. Immunoblotting

Cells were lysed in 50 mM Tris–HCl (pH 7.5), 150 mM NaCl, 1% (*w*/*v*) NP-40, and a fresh protease inhibitor cocktail (1/20, #P8340, Sigma-Aldrich). Protein quantification was performed with the BCA kit using BSA as a standard. Cell lysates (40 μg) in the SDS sample buffer were subjected to electrophoresis on a polyacrylamide gel and transferred to PVDF membranes. The membranes were incubated for 1 h with TBST containing 5% (*w*/*v*) skimmed dried milk powder or 2% (*w*/*v*) BSA. The membranes were immunoblotted in the same buffer for 16 h at 4 °C with the different primary antibodies diluted 1/1000. The blots were then washed with TBST and incubated for 1 h at room temperature with secondary HRP-conjugated antibodies diluted 2500-fold in 5% (*w*/*v*) skimmed milk in TBST. The signal was detected with enhanced chemiluminescence reagent and ChemiDoc XRS, Bio-Rad, Hercules, CA, USA.

The primary antibodies used were NLRP1 N-term (#AF6788, R&D Systems and #67980, Biolegend, San Diego, CA, USA), NLRP1 C-term (#ab36852, Abcam, Cambridge, UK), CASP1 (#sc-514, Santa Cruz), and β-ACTIN (#sc-47778, Santa Cruz Biotechnology, Santa Cruz, CA, USA). The secondary antibodies used were anti-sheep (31480, Thermofisher), anti-rabbit (#A6154, Sigma-Aldrich), and anti-mouse (#A4416, Sigma-Aldrich).

### 2.6. Evaluation of ASC Oligomerization by Flow Cytometry

Blood cells were treated with MCC950 (10 μM) or PLX-4720 (10 μM) for 30 min before stimulation with anisomycin (1 μM) for 3 h. After treatment, erythrocytes were lysed under mild hypotonic conditions while preserving and fixing leukocytes with FACS lysing/fixing solution (#349202, BD Biosciences, San Jose, CA, USA). Monocytes were stained and gated with fluorescein (FITC)-conjugated mouse anti-CD14 monoclonal antibody (clone M5E2; catalog #557153; BD Biosciences, 1:10). The detection of ASC specks in monocytes was performed by Time-of-Flight Inflammasome evaluation technique, as described [23,24] using phycoerythrin (PE)-conjugated mouse anti-ASC monoclonal antibody (HASC-71 clone; #653903; BioLegend, 1:500) and the FACS-Canto flow cytometer (BD Biosciences).

### 2.7. Next-Generation Sequencing (NGS)

Blood samples were extracted at HCUVA with the signed consent from the patient, and analysis was performed externally.

### 2.8. Evaluation of ASC Oligomerization by Fluorescent Microscopy

HEK293 ASC-GFP cells were transfected with control, wild-type NLRP1, or p.V939M NLRP1 expression plasmids using Lipofectamine 2000 (Thermo Fisher Scientific, Waltham, MA, USA). Twenty-four hours after transfection, cells were stained with Hoechst dye (H3570, Invitrogen, Waltham, MA, USA) and imaged using a Leica DMi8 fluorescence microscope. Images were acquired at 20× magnification to visualize nuclei (blue fluorescence) and ASC specks (green fluorescence). Quantification was performed using Fiji software (ImageJ 1.54g; NIH, USA). After applying a fluorescence threshold, the total number of cells was determined using the particle analyzer tool, whereas ASC specks were manually quantified using the multipoint tool. The percentage of ASC speck-positive cells was calculated relative to the total number of cells analyzed.

### 2.9. Erythroid Colony-Forming Unit (CFU-E) Assay

PBMCs (1 × 10^5^ cells) were cultured in 1 mL of MethoCult™ Methylcellulose-Based Media (Stemcell, Vancouver, BC, Canada) in the presence of CASP1 inhibitor VX-765 (100 μM, HY-13205 MedChemExpress), Ac-YVAD-cmk (37 μM inh-yvad Invivogen, San Diego, CA, USA), Ferrostatin-1 (1 μM HY-100579 MedChemExpress) or DMSO as a control. Cells were cultured at 37 °C in a humidified atmosphere with 5% CO_2_. Erythroid colony-forming units (CFU-E) were counted after 14 days of culture.

### 2.10. Flow Cytometry

Freshly prepared cell suspensions (100.000 cells) were stained using anti-CD71-FITC (14-0719-82, Invitrogen) and anti-GYPA-PE (MA-12484, Invitrogen) for 30 min in the dark. Cells were washed with 5% BSA in PBS and analyzed by FACS (BD LSR Fortessa X-20, BD Biosciences) on different days of differentiation.

### 2.11. Ferroptosis Induction Assay

K562 cells were transfected with the indicated plasmids using the 4D-Nucleofector system (Lonza, Basel, Switzerland) according to the manufacturer’s instructions and the SF Cell Line 4D-Nucleofector™ X Kit protocol. After transfection, cells were treated with hemin (10 μM) in combination with either cumene hydroperoxide (20 μM; 247502, Sigma-Aldrich) or erastin (10 μM; #17754, Cayman Chemical, Ann Arbor, MI, USA). Cells were collected at 24 h post-treatment, centrifuged, washed with PBS, and stored at −80 °C until immunoblot analysis.

### 2.12. Statistical Analyses

Data were analyzed using GraphPad Prism version 8 (GraphPad Software). Bars represent mean ±  SEM. Statistical analyses were performed using an unpaired *t*-test for comparisons between two groups, ordinary one-way ANOVA followed by Sidak’s multiple comparisons test for experiments involving more than two groups and a single variable, and two-way ANOVA followed by Sidak’s multiple comparisons test for experiments involving two independent variables.

## 3. Results

### 3.1. NLRP1 Inflammasome Is Activated in a Patient with SLC25A38-Associated CSA

**Figure 1:**
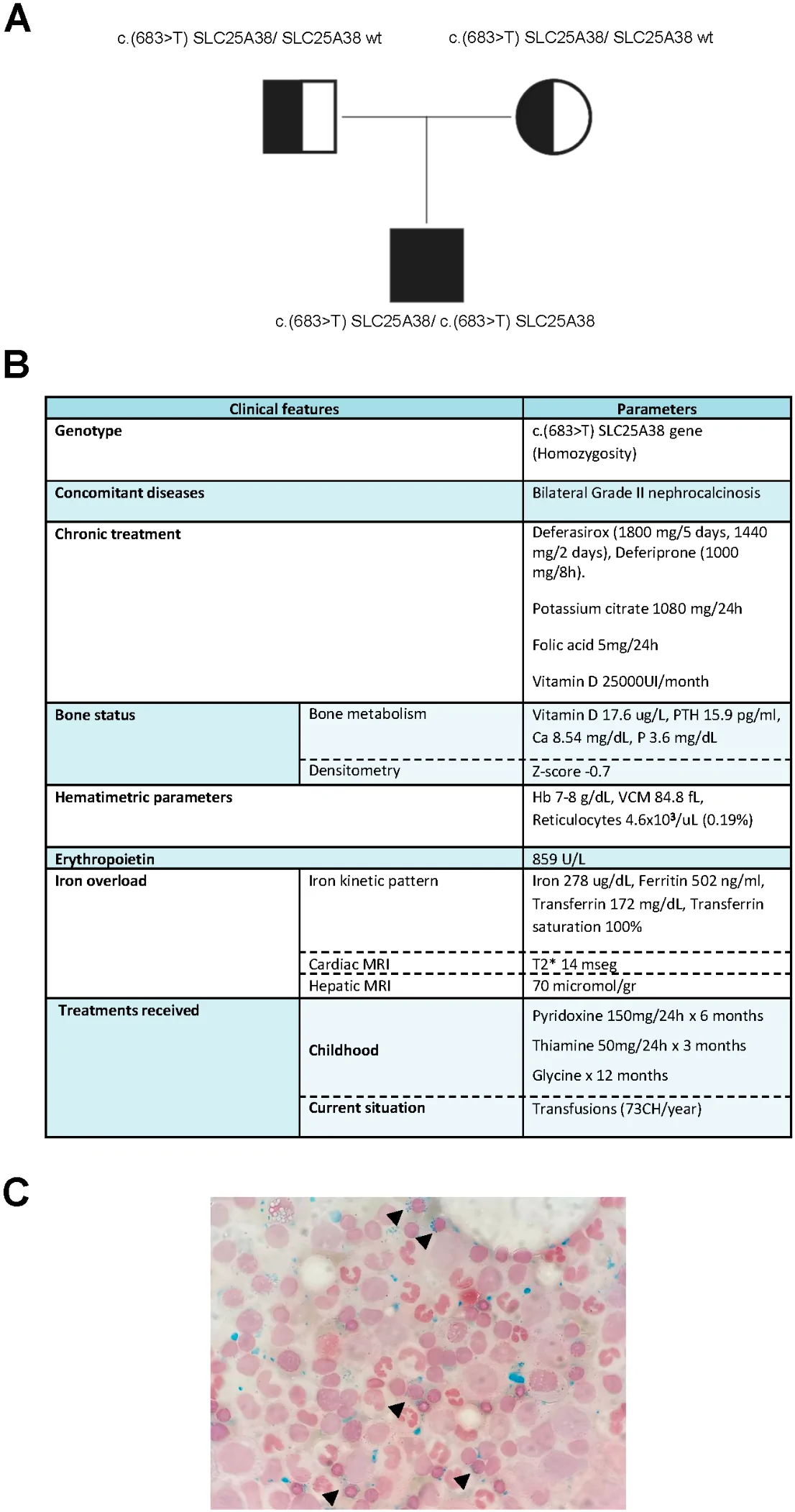
Genotypic and phenotypic characterization of the CSA patient. (**A**) Inheritance pattern of the SLC25A38 mutation. (**B**) Clinical features of the CSA patient. T2* denotes the effective transverse relaxation time measured by MRI. (**C**) Representative image of ring sideroblasts in the bone marrow of a CSA patient. Black arrows indicate ring sideroblasts.

In this study, we investigated a 20-year-old man diagnosed during infancy with CSA carrying a homozygous SLC25A38 variant (c.683G>T; p.Gly228Val), inherited from both parents (Figure 1A). The frequency of this variant is particularly rare, as reflected in the study by [25]. The patient was found to be wild-type for *ALAS2*, *SLC19A2*, *GLRX5*, and *ABCB7* genes, with no pathogenic variants identified in these genes. This patient was selected for study due to the severity of his clinical phenotype and the lack of effective curative therapies for this extremely rare disorder. Despite continuous vitamin supplementation since infancy, no hematological improvement was observed (Figure 1B). The patient currently requires chronic transfusion support consisting of three units of packed red blood cells every two weeks to maintain baseline hemoglobin levels between 7 and 8 g/dL on every visit. The patient has well-controlled iron and cardiac iron overload, as assessed by hematological parameters and cardiac MRI, with values of 44.3 mmol/g and 24 ms, respectively, and no other reported complications. Pre-transfusion hemoglobin levels range from 60 to 70 g/L, while serum ferritin is 500–850 ng/mL and transferrin saturation is 100%.

The disease course has been further complicated by several secondary manifestations, including bilateral grade II nephrocalcinosis treated with potassium citrate, hepatic iron overload, and mild left ventricular systolic dysfunction. These complications are currently managed with dual oral iron chelation therapy and cardiac supportive treatment (Figure 1B). Bone marrow examination revealed the presence of characteristic ring sideroblasts associated with mitochondrial iron accumulation (Figure 1C).

**Figure 2:**
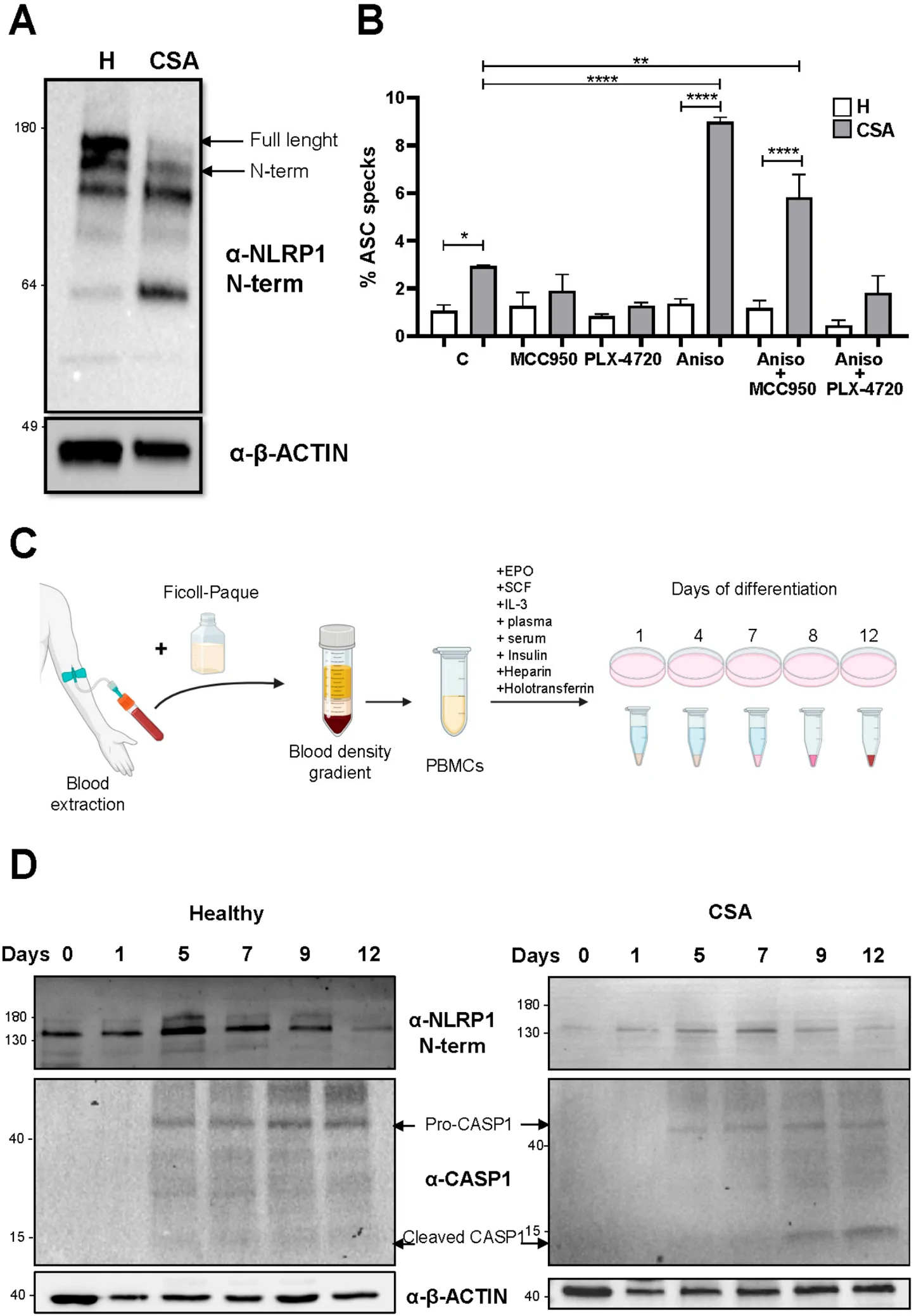
Study of NLRP1 inflammasome status in the CSA patient. (**A**) NLRP1 expression in healthy (H) and CSA patient PBMCs. (**B**) ASC specks (%) in H and CSA patient PBMCs after different treatments. (**C**) Erythroid differentiation in liquid media workflow. Blood cells were purified by density gradient to obtain PBMCs. Cells were treated with different cytokines to stimulate erythroid differentiation, and they were collected at different time points of maturation. (**D**) NLRP1, CASP1, and ACTIN immunoblots at different stages of differentiation in healthy or CSA PBMCs. Data are shown as the means  ±  SEM. *p*-values were calculated using one-way ANOVA and Sidak’s multiple range test. * *p*  <  0.05; ** *p*  <  0.01; **** *p*  <  0.0001. Western blot images are representative of three independent experiments.

Given the severity and transfusion dependence of the disease, we sought to identify molecular mechanisms that may contribute to its pathophysiology beyond the described mutation in the SLC25A38 gene. As previous studies have suggested a role for NLRP1 in erythropoiesis [20,21], we examined its expression in this patient. Strikingly, immunoblot analysis of peripheral blood mononuclear cells (PBMCs) using an N-terminal NLRP1 antibody revealed that most detectable NLRP1 protein was present in its processed form, whereas full-length NLRP1 predominated in healthy donor cells (Figure 2A).

Moreover, ASC oligomerization was observed in CD14^+^ monocytes from the patient under basal conditions, indicating constitutive NLRP1 inflammasome activation (Figure 2B). Upon anisomycin treatment, a potent, well-described inducer of NLRP1 activation, ASC speck formation was markedly enhanced in patient-derived monocytes compared to healthy donor cells. Importantly, this response was not significantly affected by MCC950, a selective inhibitor of NLRP3 [26], whereas treatment with PLX-4720, a well-characterized inhibitor of anisomycin-induced NLRP1 activation [11], significantly reduced ASC speck formation in primary cells of the patient (Figure 2B). Together, these findings strongly support the presence of abnormal NLRP1 inflammasome activation in this patient.

To explore this hypothesis further, we performed an in vitro liquid erythrocyte differentiation of an ex vivo patient sample (Figure 2C). By using Western blot analysis with an NLRP1 N-terminal antibody, we confirmed that in the patient samples, NLRP1 was fully processed during the whole differentiation (Figure 2D). Furthermore, we found evidence of CASP1 activation, as indicated by the accumulation of the p10 fragment during erythrocyte differentiation in the CSA patient samples (Figure 2D), a finding consistent with NLRP1 inflammasome activation. Nevertheless, although clear CASP1 cleavage and NLRP1 processing were observed in patient-derived stem cells undergoing erythroid maturation, together with ASC oligomerization following NLRP1 activation, these findings may also reflect, at least in part, systemic inflammation in the patient rather than erythroid cell-intrinsic mechanisms.

### 3.2. A Novel NLRP1 Variant Identified in a Congenital Sideroblastic Anemia Patient Exhibits Increased Activity Compared to Wild-Type NLRP1

**Figure 3:**
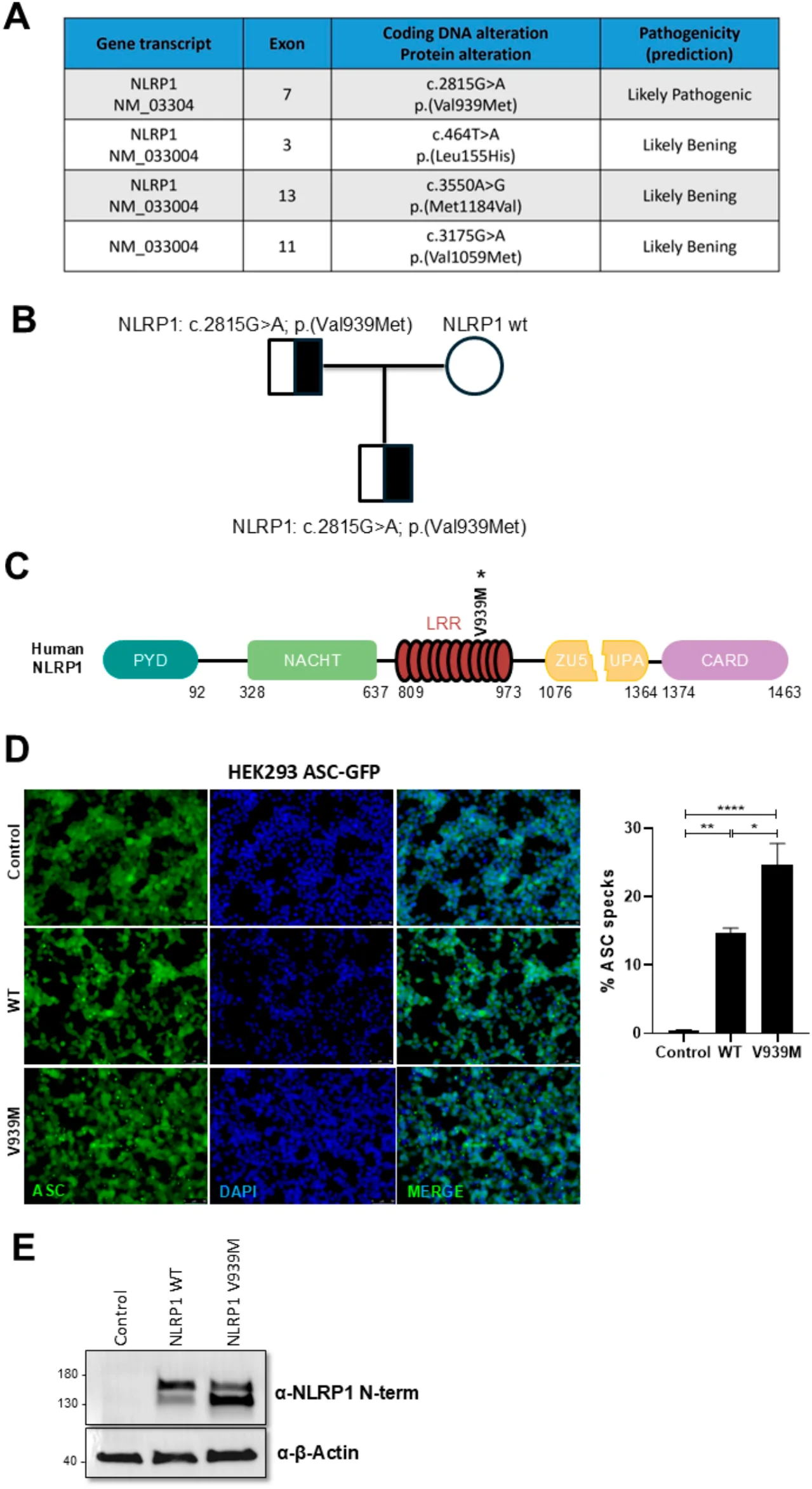
Identification of a novel gain-of-function V939M NLRP1 variant in a CSA patient. (**A**) NLRP1 variants identified by NGS analysis in a CSA patient. (**B**) Inheritance pattern of the V939M NLRP1 variant. (**C**) Localization of the V939M variant within the human NLRP1 structure. (**D**) Percentage of ASC specks relative to the total number of HEK293 ASC-GFP cells transfected with WT NLRP1 or the V939M NLRP1 variant 24 h post-transfection. (**E**) NLRP1 expression in HEK-293 ASC-GFP transfected cells from experiment (**D**). Data are shown as the mean  ±  SEM. Experiments were performed in triplicate. *p*-values were calculated using one-way ANOVA and Sidak’s multiple range test. * *p*  <  0.05; ** *p*  <  0.01; **** *p*  <  0.0001.

To investigate the underlying contribution of the increased NLRP1 inflammasome activity observed in the patient, we performed whole-exome sequencing (WES) using Next Generation Sequencing (NGS) technology and confirmed previous data on the non-pathogenic variants in *ALAS2*, *SLC19A2*, *GLRX5*, and *ABCB7* genes in this patient, as well as the known pathogenic variant in SLC25A38 (c.683G>T; p.Gly228Val). Moreover, this analysis identified four variants in the *NLRP1* gene. Three of them were present in the reference population and classified as likely benign, while one variant—c.2815G>A (p.V939M)—was predicted to be likely pathogenic (Figure 3A). Segregation analysis revealed that the patient’s father is a heterozygous carrier of the p.V939M variant (Figure 3B). The localization of this variant within the LRR domain of the NLRP1 protein is illustrated in Figure 3C.

To assess the functional consequences of this variant, we generated and expressed the NLRP1 p.V939M construct in HEK293 cells stably expressing ASC-GFP. Compared to wild-type NLRP1, the p.V939M variant induced significantly higher ASC oligomerization (Figure 3D). Western blot analysis confirmed accumulation of processed NLRP1 in the patient variant compared with wild-type and comparable protein expression levels between both constructs, excluding expression differences as the cause of enhanced inflammasome activation (Figure 3E).

### 3.3. Inhibition of CASP1 Increases Erythrocyte Formation in the Congenital Sideroblastic Anemia Patient

**Figure 4:**
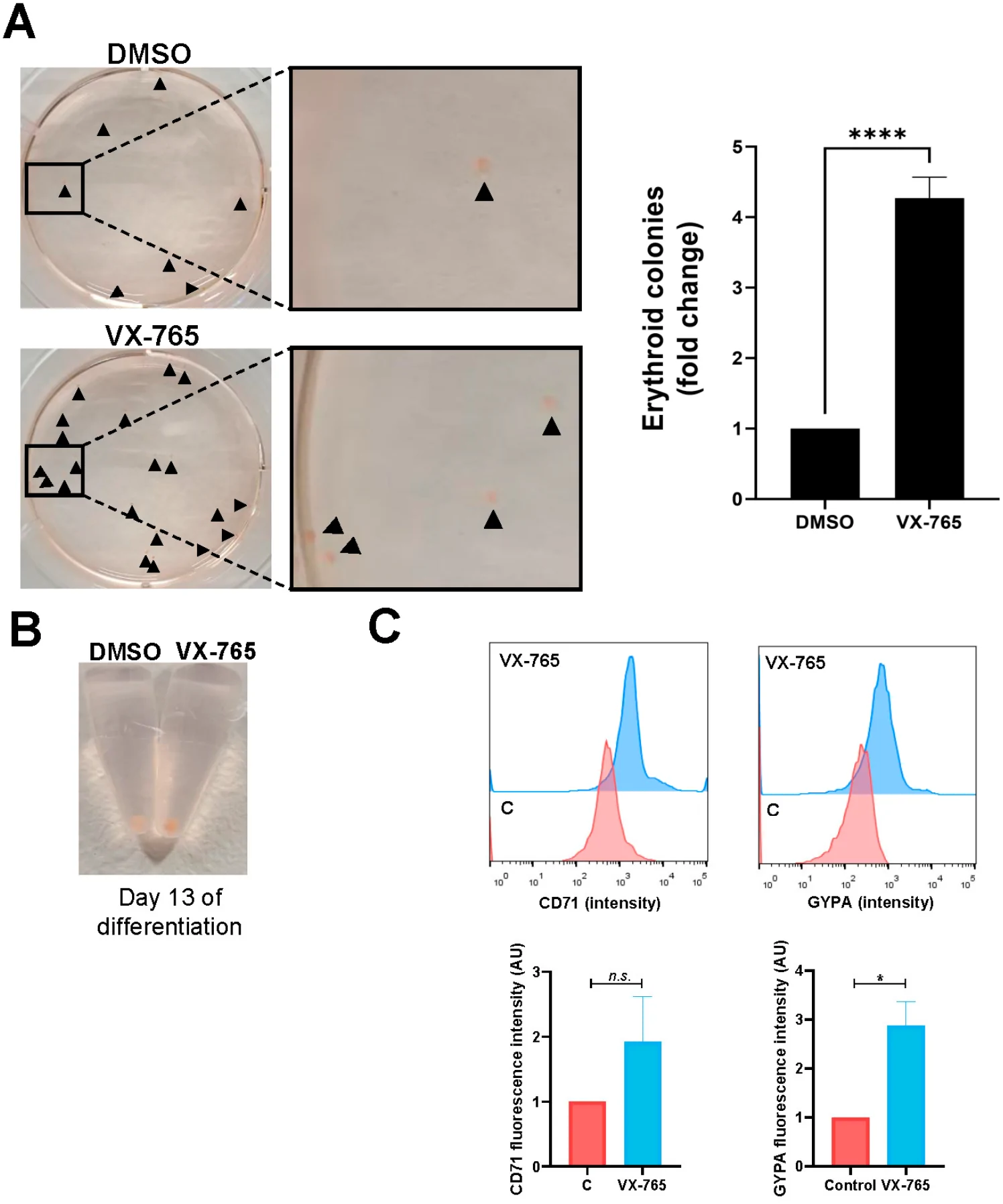
CASP1 inhibition stimulates erythroid differentiation in CSA patient cells. (**A**) Number of erythroid colonies derived from CSA PBMCs in the presence of DMSO or VX-765 on 3 different days. Erythroid colonies are indicated by black arrows. (**B**) Representative picture of CSA PBMCs at day 13 of differentiation from 3 different experiments. (**C**) CD71- and GYPA-positive cells at day 7 of differentiation in CSA PBMCs. Data are shown as the mean  ±  SEM. Experiments were performed on 3 different days with the patient sample. *p*-values were calculated using a *t*-test. n.s., non-significant; * *p*  <  0.05; **** *p*  <  0.0001.

Considering the previously described effect of CASP1 inhibition in other congenital anemias such as Diamond–Blackfan anemia and sickle cell disease [27,28] we explored the potential therapeutic value of inflammasome inhibition in this extremely rare disease. To this end, we performed erythroid colony differentiation assays using the patient’s PBMCs in the presence of the CASP1 inhibitor VX-765. CASP1 inhibition led to an increased number of erythroid colonies (Figure 4A). Furthermore, after 13 days of differentiation, CASP1 inhibition promoted hemoglobin accumulation in patient-derived cells (Figure 4B), which is consistent with the upregulation of the Glycophorin A (GYPA) marker of mature erythrocyte differentiation detected on day 7 of differentiation (Figure 4C).

### 3.4. Ferroptosis Contributes to the Processing of NLRP1 and the Severity of the Sideroblastic Anemia Phenotype

**Figure 5:**
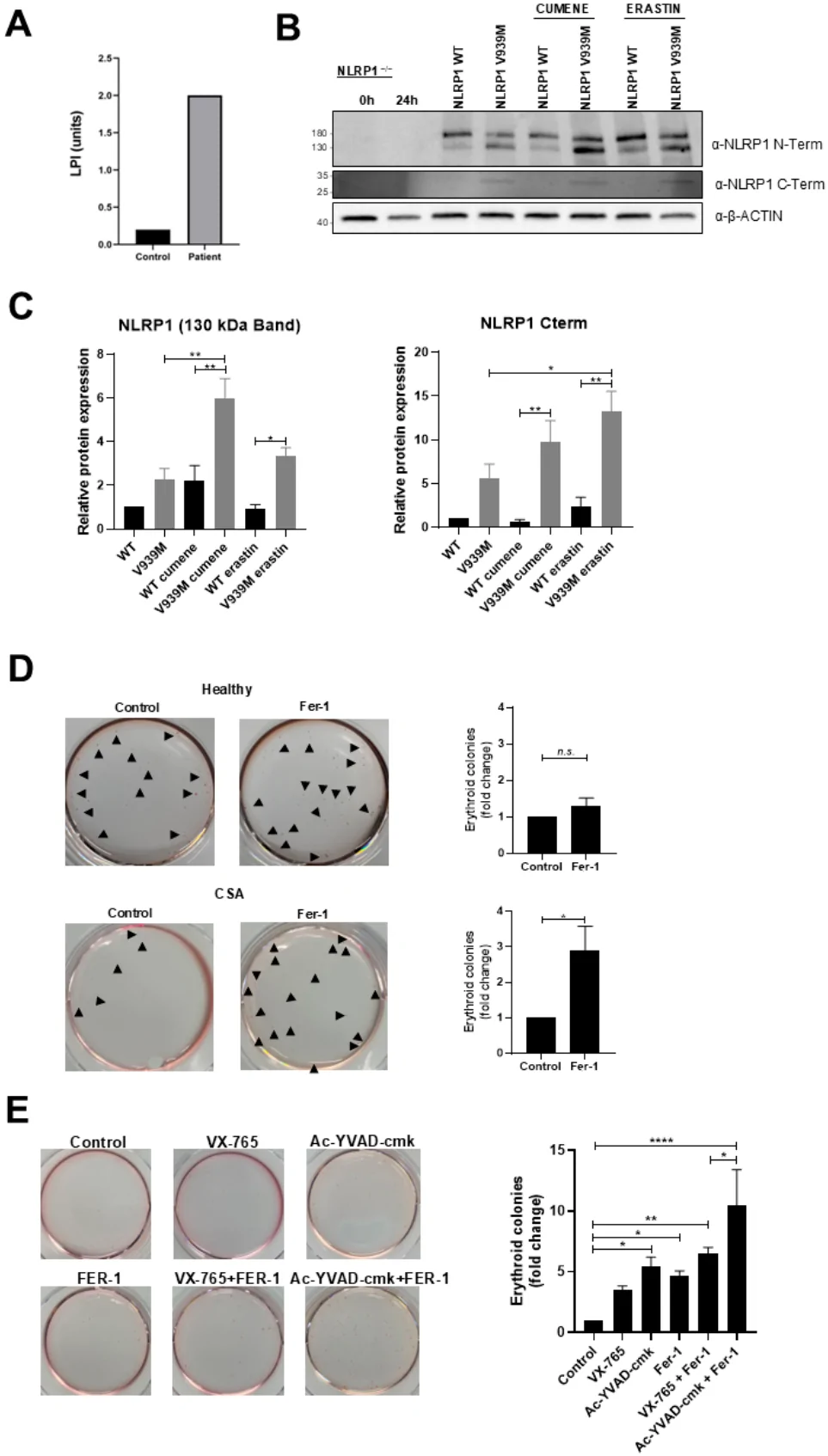
Ferroptosis and CASP-1 inhibition exert an additive effect on erythrocyte production. (**A**) Labile plasma iron (LPI) content in healthy and CSA patient blood serum. (**B**) NLRP1 and ACTIN immunoblots in NLRP1-deficient K562 cells (NLRP1^−/−^) transfected with WT or V939M NLRP1 variant and treated with cumene or erastin for 24 h in the presence of hemin. (**C**) Quantification of the 130 kDa band relative to the 170 kDa NLRP1 N-terminal band, and of the C-terminal NLRP1 signal relative to actin, by Western blot. (**D**) Number of erythroid colonies derived from healthy donors (3 independent individuals) or CSA (3 independent days) PBMCs after Fer-1 treatment. Erythroid colonies are indicated by black arrows. (**E**) Number of erythroid colonies derived from CSA PBMCs after treatment with CASP1 inhibitors (VX-765 or Ac-YVAD-cmk) and/or Fer-1. Data are shown as the mean ±  SEM. Experiments were performed with at least three independent healthy donors and 3 independent days with the CSA patient. *P*-values were calculated using a *t*-test (**D**) or one-way ANOVA and Sidak’s multiple range test (C and E). n.s., non-significant; * *p*  <  0.05; ** *p*  <  0.01; **** *p*  <  0.0001.

Considering the patient’s phenotype and clinical features such as iron overload, we determined the labile plasma iron (LPI) in the patient, which represents a component of non-transferrin-bound iron. As reflected in Figure 5A, LPI levels in the patient were approximately 10-fold higher than those detected in healthy controls and markedly exceeded the normal clinical threshold (<0.2 units). Given that excess free iron is a potent inducer of ferroptosis, we next investigated whether ferroptotic stress could differentially modulate wild-type and p.V939M NLRP1 activity. To address this question, we generated a CRISPR-Cas9 NLRP1 knockout in K562 to further study the role of human NLRP1 in erythroid differentiation. After designing the guides against exon2 of human NLRP1, we electroporated and screened the pools as described in the Methods Section and Figure S1A–C. Selected pools were sorted to single-cell clones, followed by puromycin selection to identify positive clones of this depletion. We found two clones that lacked NLRP1 protein expression (Figure S1D), and then we corroborated gene truncation by sequencing.

Using the new NLRP1 knockout cell line, we found higher NLRP1 auto-processing in the variant p.V939M compared to the wild-type, and this processing increased after ferroptosis induction by cumene or erastin treatments (Figure 5B,C). In addition, we observed an NLRP1 C-terminal active fragment (UPA-CARD) only for the p.V939M variant, which was slightly increased with ferroptosis induction (Figure 5B,C).

We therefore hypothesize that both altered NLRP1 activity and ferroptosis may contribute to defective erythropoiesis caused by the mutation in SLC25A38 in this CSA patient. To elucidate this, we performed ex vivo erythroid differentiation of patient-derived PBMCs in the presence of ferrostatin-1, a potent inhibitor of ferroptosis. Inhibition of ferroptosis increased erythrocyte production in patient-derived cells, whereas no significant effect was observed in cells from a healthy donor (Figure 5D). Furthermore, combined inhibition of ferroptosis and CASP1 using ferrostatin-1 together with VX-765 or Ac-YVAD-cmk further enhanced erythrocyte production in patient samples, suggesting an additive contribution of ferroptosis and inflammasome signaling to impaired erythropoiesis in this CSA patient (Figure 5E).

## 4. Discussion

CSA is an extremely rare and clinically heterogeneous disorder characterized by ineffective erythropoiesis and mitochondrial iron accumulation [29]. Current therapeutic approaches mainly rely on supportive management, including transfusions, iron chelation, and vitamin supplementation, but do not specifically target the molecular mechanisms underlying disease progression [30]. In Spain, we are aware of only one additional patient with SLC25A38-associated CSA, who ultimately required hematopoietic stem cell transplantation, highlighting both the rarity and severity of this condition [31]. The patient described in this study presented a particularly severe phenotype with chronic transfusion dependence, systemic iron overload, and cardiac complications despite intensive supportive treatment. These observations prompted us to investigate whether additional molecular pathways could contribute to disease severity.

In this context, we identified a novel heterozygous NLRP1 variant, p.V939M, in the patient. Several variants of NLRP1 have been associated with health disorders [32,33,34], although none have previously been linked to erythropoiesis disorders. Most pathogenic variants described to date are located within the PYD, NACHT, and UPA domains, whereas only one gain-of-function variant affecting the LRR domain, p.L813P, has previously been reported [35]. In addition, deletions involving the LRR region have been associated with familial keratosis lichenoides chronica (FKLC), likely through disruption of NLRP1 autoinhibition and consequent inflammasome hyperactivation [36]. Consistent with these observations, the p.V939M variant identified in our patient enhanced ASC oligomerization and increased NLRP1 processing, supporting a gain-of-function effect within the LRR domain.

Although the available evidence does not support classifying the p.Val939Met (p.V939M; NM_014922.4.2815G>A; rs61754791) variant as pathogenic on the basis of this variant alone, evidence from ClinVar, OMIM, and Franklin by Genoox supports its classification as a variant of uncertain significance (VUS), and the presence of this variant in the general population, including in one of the progenitors described in this case report who did not exhibit any reported clinical symptoms, confirms that it has no direct pathogenic effect. In any case, our findings raise the possibility that the phenotypic impact of p.Val939Met may be influenced by additional genetic or cellular factors, particularly those involving ferroptosis-related pathways. In the context of sideroblastic anemia, this variant may potentially act as a disease modifier, contributing to or exacerbating the clinical phenotype in the presence of other pathogenic variants or susceptibility factors. Further functional and clinical studies are needed to establish the biological significance of p.Val939Met and to determine whether its interaction with ferroptosis-related mechanisms contributes to the development or severity of the sideroblastic anemia phenotype. It has been described that human NLRP1 needs to be processed to liberate the C-terminal active fragment [18,19]. Under resting conditions, this fragment remains bound to the full-length protein in an inactive complex stabilized by DPP9 [19]. Nevertheless, it has been demonstrated that DPP9 and NLRP1 do not interact in human K562 cells [21], suggesting an alternative regulatory mechanism in hematopoietic cell lines. In agreement with this possibility, we observed constitutive NLRP1 processing in PBMCs derived from the patient, together with increased ASC speck formation and enhanced CASP1 activation during erythroid differentiation. Collectively, these findings support the presence of sustained inflammasome signaling in patient-derived hematopoietic cells and suggest that aberrant NLRP1 activation may contribute to ineffective erythropoiesis in severe CSA.

Moreover, a central finding of this study is the association between ferroptotic stress and NLRP1 hyperactivation. Ferroptosis is a form of regulated cell death driven by iron-dependent lipid peroxidation and reactive oxygen species accumulation [37,38]. Given that sideroblastic anemia is characterized by pathological iron accumulation, we hypothesized that ferroptosis could potentiate inflammasome activation in this patient. Supporting this possibility, the patient exhibited markedly elevated levels of labile plasma iron. Furthermore, induction of ferroptotic/oxidative stress using cumene or erastin enhanced NLRP1 processing in K562 cells expressing the p.V939M variant compared with wild-type NLRP1 in a model lacking endogenous NLRP1, suggesting that the mutant protein is particularly sensitive to ferroptotic stress.

Importantly, our ex vivo functional studies indicate that both ferroptosis and inflammasome activation contribute to defective erythropoiesis in this patient. Notably, simultaneous inhibition of ferroptosis and CASP1 resulted in an additive effect, suggesting crosstalk between iron-driven oxidative stress and inflammasome activation in CSA. Although the precise molecular link between ferroptosis and NLRP1 activation remains to be fully elucidated, our findings support a model in which iron overload creates a ferroptotic environment that enhances activation of the p.V939M NLRP1 variant, thereby aggravating ineffective erythropoiesis.

Although limited to a single patient, our case report study identifies a previously unreported NLRP1 inflammasome variant with enhanced activity compared with the wild-type protein, suggesting a potential role as a genetic modifier of disease severity in SLC25A38-associated congenital sideroblastic anemia (CSA). The discovery of the gain-of-function p.V939M NLRP1 variant, together with the observed improvement in erythropoiesis following pharmacological inhibition of ferroptosis and CASP1 in patient-derived cells, supports the involvement of these pathways in disease pathogenesis. These findings further suggest that targeted modulation of inflammasome activation and ferroptotic cell death may represent a personalized therapeutic approach for a subset of patients with CSA.

## Data Availability

The raw data supporting the conclusions of this article will be made available by the authors on request.

## Supplementary Materials

The following supporting information can be downloaded at: https://www.mdpi.com/article/10.3390/jpm16090471/s1. Figure S1: Generation and validation of NLRP1 knockout clones in K562 cells. (A) NLRP1 knockout (NLRP1-/-) cell line generation by CRISPR workflow. (B) Schematic representation of the NLRP1 gene, highlighting exon 2, which is common to all NLRP1 isoforms and was selected as the CRISPR/Cas9 targeting region. (C) Western blot analysis of NLRP1 expression following electroporation with the different guide RNA pairs described in Materials and Methods. Based on these results, pool 2.2 was selected for sorting and subsequent expansion of individual clones under puromycin selection. (D) Western blot analysis of the resulting individual clones identified two NLRP1 knockout (KO) clones, which were selected for further experiments.
