# Supplementary material for: A New Gain-of-Function NLRP1 Variant Is Associated with Inflammasome Activation and Disease Severity in SLC25A38 Congenital Sideroblastic Anemia Case Report
Source: J Pers Med. 2026 Sep 15;16(9):471. doi: 10.3390/jpm16090471 (PMC13608223; doi:10.3390/jpm16090471)
Supplement: Supplementary file 1 [file jpm-16-00471-s001.zip › jpm-4458890-supplementary.pdf]

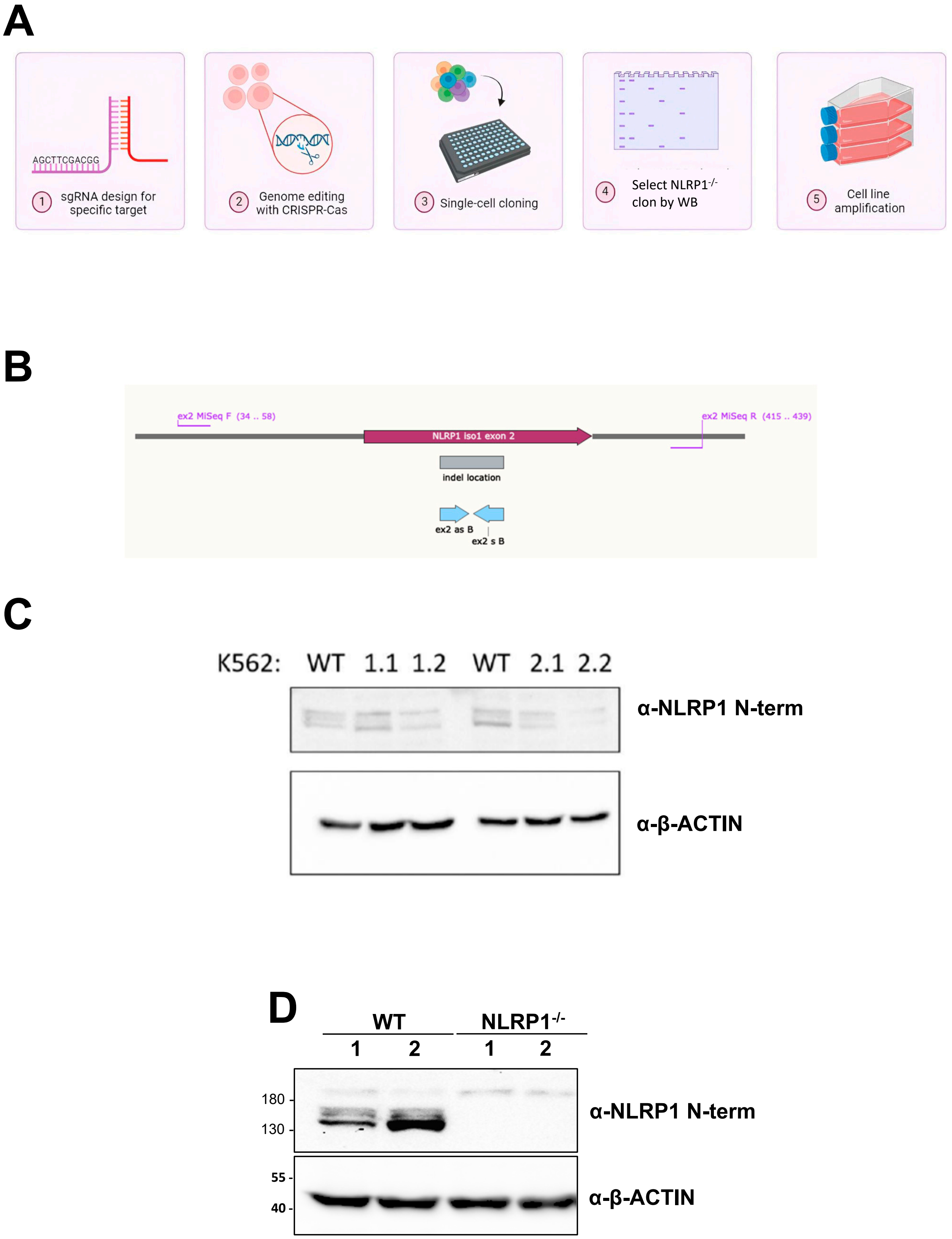
A
AGCTTCGACGG
1 sgRNA design for specific target
2 Genome editing with CRISPR-Cas
3 Single-cell cloning
4 Select NLRP1-/- clon by WB
5 Cell line amplification
B
ex2 MiSeq F (34 .. 58)
ex2 MiSeq R (415 .. 439)
NLRP1 iso1 exon 2
indel location
ex2 as B
ex2 s B
C
K562: WT 1.1 1.2 WT 2.1 2.2
α-NLRP1 N-term
α-β-ACTIN
D
WT
NLRP1-/-
1 2 1 2
180
130
55
40
α-NLRP1 N-term
α-β-ACTIN

Figure S1
